# Starch intake and changes in dental caries among adults: A longitudinal study in Finland

**DOI:** 10.1111/jphd.12650

**Published:** 2024-11-16

**Authors:** F. H. Jangda, A. L. Suominen, A. Lundqvist, S. Männistö, A. Golkari, E. Bernabé

**Affiliations:** ^1^ Institute of Dentistry Queen Mary University of London London UK; ^2^ Institute of Dentistry University of Eastern Finland Kuopio Finland; ^3^ Oral Health Teaching Unit Kuopio University Hospital Kuopio Finland; ^4^ National Institute for Health and Welfare Helsinki Finland

**Keywords:** adults, dental caries, plant roots, seeds, solanum tuberosum, starch

## Abstract

**Objective:**

To evaluate the association between starch intake (amount and type) and changes in dental caries among adults over 11 years.

**Methods:**

Data from 1679 adults, aged 30 years and over, who participated in three consecutive surveys in Finland were pooled for analysis. Participants completed a validated semi‐structured 128‐item food frequency questionnaire at baseline, from which total starch intake (g/day and % energy intake) and the intake (g/day) of seven food groups high in starch (potatoes, potato products, roots and tubers, refined grains, pasta, wholegrains, and legumes) were estimated. Dental caries was determined during clinical examinations and summarized using the DMFT score, which was treated as a repeated outcome. The association between baseline starch intake and 11‐year‐change in DMFT score was tested in linear mixed‐effects models adjusted for sociodemographic factors, behaviors, sugar intake, and health status.

**Results:**

The mean DMFT score was 21.9 (95%CI: 21.6, 22.2) in 2000 (baseline), increasing by 0.47 (95% CI: 0.38, 0.56) in 2004/05, and additionally by 0.33 (95%CI: 0.20, 0.45) in 2011. Total starch intake was not associated with change in DMFT. This finding was similar irrespective of how starch intake was expressed (g/day or %EI). Of the seven food groups evaluated, only the intake of pasta was inversely associated with the DMFT score at baseline, but not with the change in DMFT over time.

**Conclusion:**

Neither the amount nor the type of starch intake was associated with changes in dental caries over 11 years among Finnish adults.

## INTRODUCTION

Starch is the predominant carbohydrate in human diet, providing the body with energy [1, 2]. It is the storage carbohydrate of plants such as cereals, root vegetables, and legumes and consists of only glucose molecules [1]. As such, it is a significant source of glucose in the human diet and plays a major role in post‐meal variations in blood sugar levels [3, 4]. Based on their resistance to human digestive enzymes starches are classified as rapidly digestible (RDS), slowly digestible (SDS), and resistant (RS) [5]. RDS is digested quickly in the small intestine (converted to glucose in less than 20 min) and is found in freshly cooked starchy foods (i.e. boiled potatoes) and highly processed starches like breads, instant potato powder, and potato crisps. SDS is slowly digested in the small intestine (converted to glucose within 20 to 120 min), and is found in forms of pasta, nuts and seeds. RS is not digested in the small intestine but undergoes microbial fermentation in the gut producing short chain fatty acids. Common sources of RS include wholegrains and legumes [5, 6, 7]. There are no current recommendations for total starch intake. Instead, the World Health Organization (WHO) recommends that carbohydrate intake should mainly come from wholegrains, vegetables, fruits, and pulses [8]. This recommendation has been endorsed by the European Food Safety Authority (EFSA) Panel on Nutrition, Novel Foods, and Food Allergens [9], and the 2023 Nordic Nutrition recommendations [10].

Starch is not fermentable by oral bacteria and pose no direct cariogenic risk. However, it can be broken down into sugars by the action of salivary amylase as soon as it is ingested, which raises the question of whether its intake could be associated with dental caries [11, 12]. The UK's Scientific Advisory Committee on Nutrition (SACN) report on carbohydrates and health identified a lack of available evidence on the relationship between starch or starch‐rich foods and oral health outcomes [13]. A systematic review, conducted to inform the recently published WHO guideline on carbohydrate intake for adults and children [8], found low‐quality evidence (4 cohort studies, one in adults) suggesting no association between total starch intake and dental caries. The review also found low‐quality evidence (2 cohort studies among children) suggesting a positive association between RDS intake (one study focused on processed starches at snack time and the other study focused on sugar‐starch interactions in foods) and dental caries. Overall, evidence was limited, and results from the primary studies were generally not amenable to meta‐analysis [14]. Another systematic review concluded that the frequent consumption of processed sugar‐and‐starch‐containing foods between meals was associated with dental caries (3 cohort studies among children) [12]. It must be noted that most primary studies identified in the two previous reviews evaluated the combined effect of starch and sugars intake [12, 14], making it difficult to separate the independent effect of starch consumption on caries risk. Another point to note is that almost all studies were carried out among children.

There is a need for well‐conducted longitudinal studies among adults in this research area. To fill this gap in knowledge, this study will assess both total starch intake and common sources of starch (including RDS) in the Finnish diet, analyze three waves of caries data over a decade and control for various determinants of diet and dental caries. The findings of this study can inform future recommendations regarding the consumption of starch. The level of fluoride in water in Finland is low, with a fluoride concentration <0.1 mg/L in 98% of water from waterworks and 95% of single‐well waters [15]. The aim of this study was to evaluate the relationship between starch intake (in terms of amount and relevant food sources) and changes in dental caries among adults over an 11‐year period. It was hypothesized that a high intake of starch, particularly sources of RDS, would be positively associated with greater levels of dental caries.

## MATERIALS AND METHODS

### Data source

**FIGURE 1:**
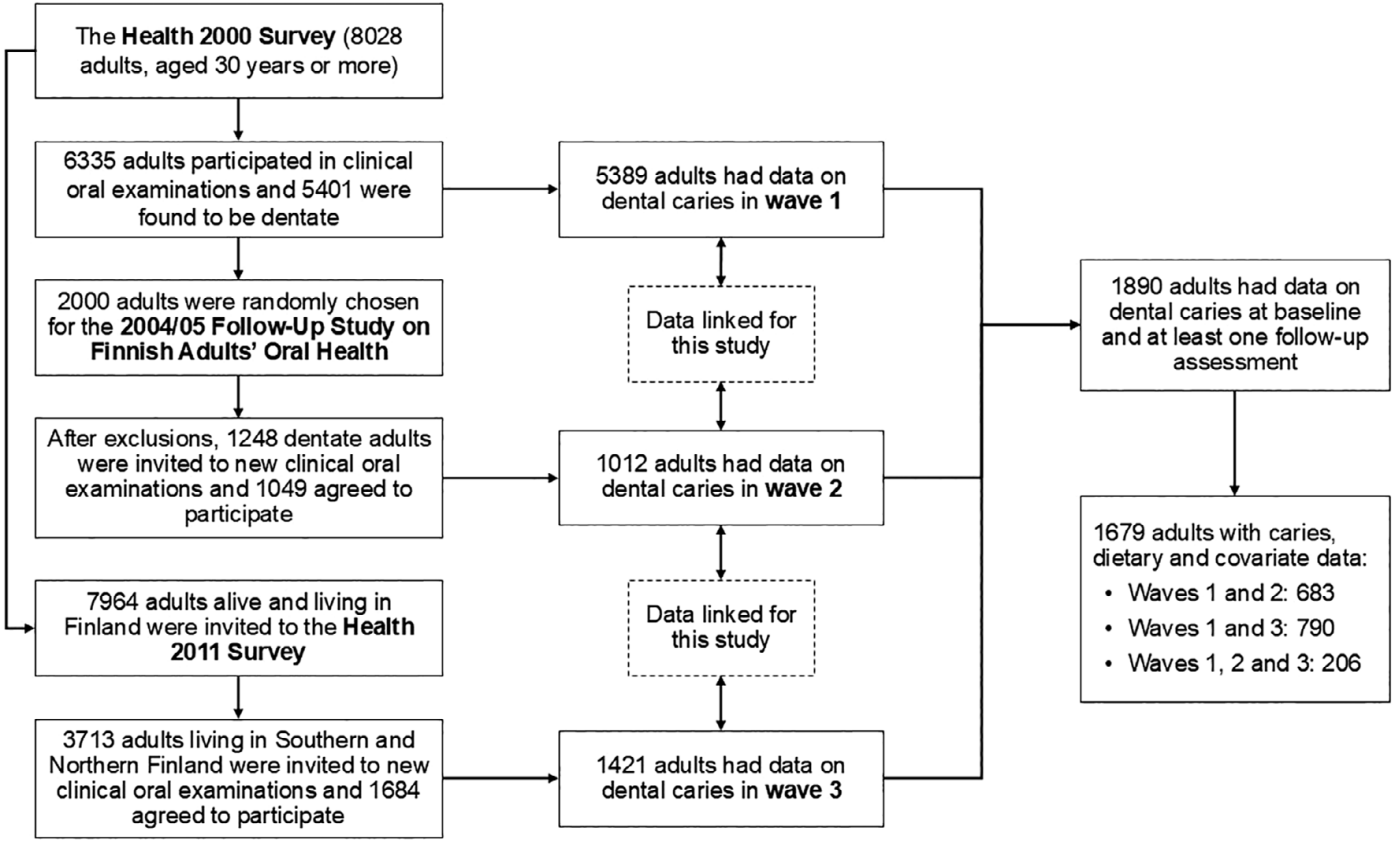
Flowchart of participation in each survey and data linkage across surveys.

This study pooled data from adults who participated in the Health 2000 survey and at least one out of two follow‐up surveys (Figure 1). The Health 2000 survey (wave 1, baseline data) recruited a national sample of 8028 adults aged 30 years or older living in mainland Finland. Of them, 6335 (79%) received dental examinations and 5401 were found to be dentate [16]. The 2004/05 Follow‐Up Study on Finnish Adults' Oral Health (wave 2) recruited 2000 adults who were randomly selected from those who underwent dental examinations in the Health 2000 survey. People who died or were edentate, and those who lived in areas where <15 participants were sampled for the Health 2000 survey were excluded. After exclusions, 1248 adults were invited for a new dental examination and 1049 agreed to participate (84%). The Health 2011 survey (wave 3) recruited 7694 participants of the Health 2000 survey alive and living in mainland Finland. Dental examinations were carried out in two of the five recruitment areas (Southern or Northern Finland), with 3713 adults invited to participate and 1684 re‐examined (45%) [17]. All surveys were carried out by the Finnish Institute for Welfare and Health (THL). Ethical approval was sought for each survey separately, from the Ethics Committee at the Hospital District of Helsinki and Uusimaa. All participants provided written informed consent.

For this study, there were 1890 dentate participants at baseline who had participated in one or both follow‐up dental examinations (Figure 1). Of them, 211 were excluded because of missing data on baseline dietary intake (*n* = 98) or covariates (*n* = 113). Thus, the final sample included 1679 participants, of which 1473 and 206 had caries data on two and three waves, respectively.

### Variables

Dental caries experience was the study outcome, which was determined from clinical dental examinations by five trained dentists. Clinical examinations were carried out following the same protocol in every wave. Participants were seated on a dental chair and clinically examined using a fiber optic light, mouth mirror, and WHO periodontal probe. Teeth were dried using compressed air and isolated with cotton rolls to facilitate visual inspection. A tooth was considered decayed if there was evidence of a carious lesion extending into dentine on any coronal or root surface, and the lesion was cavitated, penetrating the fissure and undermining the enamel, or the dentine walls were clearly softened [18]. The inter‐ and intra‐examiner reliability for caries detection at tooth level was high, with Kappa values of 0.87 and 0.95, respectively [18]. Dental caries was summarized using the number of decayed, missing and filled teeth (DMFT score) and treated as a repeated measure in analysis.

Starch intake at baseline (wave 1) was the exposure of interest. A validated semi‐structured food frequency questionnaire (FFQ) was used to measure habitual dietary intake in the past year [19, 20, 21]. The FFQ comprised of 128 commonly used or nutritionally important food items and mixed dishes, that were presented to participants in 12 sections: dairy products; cereals; spreads; vegetables; potatoes, rice, and pasta; meat; fish; chicken, turkey, and eggs; fruits and berries; desserts; sweets and snacks; and beverages. A standard portion size was assigned to each FFQ item using natural units (piece, slice, glass, tablespoon, etc.). Each FFQ item had nine response options (never or rarely, 1–3 times per month, once per week, 2–4 times per week, 5–6 times per week, once a day, 2–3 times a day, 4–5 times a day, and more than 6 times a day). The FFQ was completed at home and sent to THL. When returned, they were checked for unreliable and missing answers by a nutritionist. Responses were used to derive the intakes of starch, sugars, and food groups and energy intake [EI] using the Finnish Food Composition Database (Fineli®, THL, Finland). Total starch intake was analyzed in quintiles of amount (g/day) and percent of EI (%EI). Based on existing literature [5, 6, 7], seven common food sources of starch were included in the analysis: (i) potatoes, (ii) potato products (i.e. French fries, potato chips), (iii) roots and tubers, (iv) legumes (e.g. peas, beans, nuts, and seeds), (v) wholegrains (e.g. barley, oat, and rye) [22], (vi) pasta, and (vii) refined grains (total grains minus wholegrains and pasta, i.e. wheat, rice). All food sources were analyzed as quintiles of amount consumed (g/day). Other sources of starch (such as crackers) were not considered because they were not part of the FFQ (i.e. they are not frequently eaten by Finnish adults) and contain both starch and sugars, which would have complicated separating their effects.

We used the disjunctive cause criterion [23, 24] to select confounders of the association between starch intake and dental caries. It postulates that sufficient control for confounding can be achieved by (i) controlling for each covariate that is a cause of the exposure, or of the outcome, or of both; (ii) excluding from this set any variable known to be an instrumental variable; and (iii) including as a covariate any proxy for an unmeasured variable that is a common cause of both the exposure and the outcome. Sex, age, education, and self‐rated health were considered common causes of starch intake and dental caries. Sugar intake, toothbrushing frequency, use of fluoride toothpaste, use of interdental cleaning aids, and dental attendance were considered causes of dental caries. Marital status, physical activity, alcohol consumption, body mass index (BMI), and history of chronic conditions were considered causes of starch intake (Figure S1). Current general health status was reported on a 5‐point ordinal scale and categorized as poor, moderate, or good. Toothbrushing was reported using a 5‐point ordinal scale (more often than twice a day, twice a day, once a day, less frequently than every day and never) and categorized as twice or more daily, once daily, or less than daily. Sugar intake was analyzed in quintiles of amount (g/day). Use of fluoride toothpaste and interdental cleaning aids (i.e. flossing or interdental brushes) were reported using a 4‐point ordinal scale (daily, weekly, less frequently, not at all) and categorized as daily, less than daily, or never. Dental attendance was reported using three response options and categorized as for check‐ups or only when in trouble (including those who had never visited the dentist). Marital status was categorized as cohabiting (married and living with partner) or living alone (single, divorced or living apart and widowed). Physical activity was assessed with two questions on exercise during leisure time (LTE) for at least 30 min (so they felt at least slightly out of breath and sweating) and walking or cycling to work (WCW). Physical activity was categorized as ideal (LTE ≥4 times/week and WCW ≥ 30 min/day), sufficient (when only the LTE or WCW threshold was met), low (LTE = 2–3 times/week and WCW < 30 min/day) and sedentary (LTE ≤1 time/week and WCW < 30 min/day). Weekly alcohol intake (100% ethanol) was categorized as no use, moderate use (women<70 g, men<140 g) and risk use (women≥70 g, men≥140 g). Participants' weight and height were measured by trained nurses using a wall‐mounted stadiometer and a bioimpedance device's scale (InBody 3.0, Biospace, South Korea), respectively. Participants were classified as normal weight (BMI < 25 kg/m^2^), overweight (25–29.9), or obese (≥30). Participants also reported if they had ever been diagnosed with hypertension, diabetes, heart disease, or stroke.

### Statistical analysis

All analysis were performed in Stata MP 18 (StataCorp LP, College Station, Texas). We first compared the baseline characteristics of participants in each quintile of total starch intake (g/day) using the Chi‐square test. Then, we compared the DMFT score in every wave (2000, 2004/05, and 2011) by quintiles of total starch intake (expressed in g/day and %EI, respectively). Tests for linear trend from crude linear regression models were used for these comparisons.

A linear mixed‐effects (LME) model was fitted to model the change in DMFT score over 11 years, with the three repeated caries assessments (level 1) nested within individuals (level 2). LME models utilize all available outcome data, manage observations that are missing or unevenly distributed over time and adjust for the correlation among repeated measurements within the same individual [25, 26]. Time was modeled flexibly using a categorical indicator (wave 1 = 0, wave 2 = 1, and wave 3 = 2), with both its intercept and slope incorporated as random effects to characterize individual differences in baseline DMFT score and its rate of change over time. All other model predictors were estimated as fixed effects. The covariance matrix was estimated without any restrictions (unstructured). A model with time as the only predictor was initially fitted to determine the average rate of change in DMFT score from wave to wave. Then, the association between total starch intake at baseline and 11‐year change in DMFT score was evaluated in three sequential models. Model 1 included starch intake, EI, and time as predictors. Model 2 additionally adjusted for sociodemographic factors, health behaviors, and health condition. Model 3 also included the interaction between starch intake and time. The likelihood ratio test was used to check whether adding the interaction term improved the goodness‐of‐fit of the model. The simpler model (Model 2) was preferred if there was no evidence to reject the null hypothesis for the likelihood ratio test [25]. The estimates from Models 1 and 2 represent the effect of starch intake on the baseline DMFT score that will continue unchanged over the follow‐up period (parallel lines for the intake quintiles) whereas a significant interaction with time in Model 3 indicates that the effect of starch intake on DMFT score varies over time (divergent or convergent lines for the intake quintiles) [26]. Separate LME models were fitted for quintiles of total starch intake in g/day and %EI, respectively. The same set of three sequential LME models was fitted for each of the seven food sources of starch separately, although they were not adjusted for sugar intake.

Several supplemental analyses were conducted to evaluate our methodological decisions. First, we compare the characteristics of adults in the study sample, adults without follow‐up data on dental caries and adults with follow‐up data on dental caries but no data on diet or covariates to evaluate the impact of attrition and missing data, respectively. Second, we rerun all models using the DFT score instead of the full DMFT score to evaluate the influence of including missing teeth on the findings. Third, we rerun all models adjusting for common causes of exposure and outcome only (i.e. sex, age groups, education, and self‐rated health) to evaluate the impact of the method used for selection of confounders. Finally, we determined the power of our study to identify the observed associations.

## RESULTS

Data from 3564 repeated observations in 1679 adults were analyzed. The mean age of participants was 47.6 ± 11.3 years at baseline. The mean total starch intake was 130.2 ± 50.3 g/day, representing 23.2 ± 4.8%EI. Male, older, and less educated participants, those cohabiting and reporting more physical activity and no alcohol consumption were more likely to be in the highest quintiles of starch intake (Table 1). There were increasing linear trends in the crude DMFT score according to quintiles of total starch intake (g/day) in waves 1 and 2 but not in wave 3 (Table 2). Increasing trends in DMFT score were also noted according to quintiles of total starch intake (%EI) in every wave.

**TABLE 1:** Baseline characteristics of the study sample according to quintiles of starch intake and baseline covariates.

Baseline characteristics	All sample		Q1 (median: 71.6 g/day)		Q3 (median: 123.4 g/day)		Q5 (median: 194.0 g/day)	
	*n*	%	*n*	%	*n*	%	*n*	%
Sex								
Male	740	44.1	131	39.0	131	39.0	192	57.3
Female	939	55.9	205	61.0	205	61.0	143	42.7
Age groups								
30–39 years	498	29.7	102	30.4	100	29.8	92	27.5
40–49 years	487	29.0	98	29.2	96	28.6	104	31.0
50–59 years	423	25.2	98	29.2	72	21.4	78	23.3
60–69 years	209	12.5	34	10.1	51	15.2	46	13.7
70+ years	62	3.7	4	1.2	17	5.1	15	4.5
Education								
Basic	402	23.9	88	26.2	71	21.1	85	25.4
Secondary	678	40.4	139	41.4	160	47.6	107	31.9
Higher	599	35.7	109	32.4	105	31.3	143	42.7
Marital status								
Cohabiting	1275	75.9	225	67.0	260	77.4	272	81.2
Living alone	404	24.1	111	33.0	76	22.6	63	18.8
Physical activity								
Sedentary	611	36.4	148	44.1	114	33.9	115	34.3
Low	531	31.6	98	29.2	107	31.9	94	28.1
Sufficient	454	27.0	80	23.8	100	29.8	101	30.2
Ideal	83	4.9	10	3.0	15	4.5	25	7.5
Alcohol consumption								
No use	173	10.3	16	4.8	33	9.8	59	17.6
Moderate use	1158	69.0	222	66.1	239	71.1	230	68.7
Risk use	348	20.7	98	29.2	64	19.1	46	13.7
BMI group								
Normal	700	41.7	137	40.8	150	44.6	139	41.5
Overweight	659	39.3	130	38.7	130	38.7	130	38.8
Obese	320	19.1	69	20.5	56	16.7	66	19.7
Diabetes								
No	1630	97.1	331	98.5	323	96.1	324	96.7
Yes	49	2.9	5	1.5	13	3.9	11	3.3
Hearth disease								
No	1385	82.5	275	81.9	284	84.5	275	82.1
Yes	294	17.5	61	18.2	52	15.5	60	17.9
Hypertension								
No	1252	74.6	261	77.7	253	75.3	235	70.2
Yes	427	25.4	75	22.3	83	24.7	100	29.9
Stroke								
No	1657	98.7	333	99.1	335	99.7	328	97.9
Yes	22	1.3	3	0.9	1	0.3	7	2.1
Self‐rated general health								
Poor	82	4.9	14	4.2	17	5.1	19	5.7
Moderate	357	21.3	65	19.4	60	17.9	81	24.2
Good	1240	73.9	257	76.5	259	77.1	235	70.2
Toothbrushing								
Twice or more daily	1153	68.7	239	71.1	235	69.9	208	62.1
Once daily	459	27.3	85	25.3	89	26.5	110	32.8
Less than daily	67	4.0	12	3.6	12	3.6	17	5.1
Fluoride toothpaste use								
Daily	1555	92.6	316	94.1	306	91.1	307	91.6
Less than daily	83	4.9	17	5.1	20	6.0	17	5.1
Never	41	2.4	3	0.9	10	3.0	11	3.3
Interdental cleaning								
Daily	200	11.9	35	10.4	33	9.8	41	12.2
Less than daily	695	41.4	146	43.5	139	41.4	132	39.4
Never	784	46.7	155	46.1	164	48.8	162	48.4
Dental attendance								
For check‐ups	1055	62.8	210	62.5	205	61.0	210	62.7
Only when in trouble	624	37.2	126	37.5	131	39.0	125	37.3

*Note*: Only the first, third and fifth quintiles (Q1, Q3, Q5) are presented for simplicity.

Abbreviation: BMI, body mass index.

**TABLE 2:** Crude associations between total starch intake at baseline and DMFT score in 2000, 2004/05, and 2011 (waves 1 to 3, respectively).

Total starch intake	Wave 1 (*n* = 1679)		Wave 2 (*n* = 889)		Wave 3 (*n* = 996)	
	Mean	(SD)	Mean	(SD)	Mean	(SD)
In g/day						
Q1 (median: 71.6)	21.3	(6.5)	21.7	(5.9)	22.2	(6.0)
Q2 (101.4)	22.3	(6.2)	22.6	(5.7)	23.1	(6.1)
Q3 (123.4)	21.3	(6.7)	21.4	(6.8)	22.0	(6.4)
Q4 (150.5)	22.2	(6.0)	22.9	(5.4)	22.6	(5.8)
Q5 (194.0)	22.4	(6.3)	23.0	(6.3)	22.9	(6.3)
*p* value for trend^a^	0.047		0.039		0.584	

As %EI						
Q1 (median: 17.2%)	21.6	(6.1)	22.0	(5.8)	22.5	(5.7)
Q2 (20.7%)	21.2	(6.6)	21.6	(6.1)	21.9	(6.2)
Q3 (23.1%)	21.5	(6.3)	22.1	(5.9)	22.0	(6.4)
Q4 (25.5%)	21.9	(6.5)	22.2	(6.5)	22.7	(6.2)
Q5 (29.5%)	23.2	(6.1)	23.8	(5.8)	23.6	(6.0)
*p* value for trend^a^	0.001		0.005		0.032	

Abbreviations: %EI, percent of total energy intake; SD, standard deviation; Q, quintiles.

^a^ Tests for linear trend were derived from linear regression models.

The LME model with time as the only predictor showed that the mean DMFT score was 21.9 (95%CI: 21.6, 22.2) at baseline, increasing by 0.47 (95% CI: 0.37, 0.56) in wave 2 and additionally by 0.33 (95%CI: 0.20, 0.45) in wave 3. The negative covariance of −1.86 (95%CI: −2.27 to −1.45) indicated that the largest DMFT increments were observed among those with the lowest DMFT score at baseline. Total starch intake was positively associated with the baseline DMFT score before adjustment for covariates (Model 1, Table 3). This finding was similar irrespective of how starch intake was expressed (g/day or %EI). However, these associations were fully attenuated after adjustment for covariates (Model 2, Table 3). The interaction of each indicator of total starch intake with time was not significant in Model 3 (*p* = 0.328 for starch intake in g/day and 0.315 for starch intake as %EI), suggesting that starch intake was not associated with the 11‐year change in DMFT score.

**TABLE 3:** Models for the association between starch intake and 11‐year change in DMFT among Finnish adults 30 years and older (3564 observations in 1679 participants).

Starch intake	Model 1		Model 2	
	Coef.	(95% CI)	Coef.	(95% CI)
In g/day				
Q1 (median: 71.6)		Reference		Reference
Q2 (101.4)	1.09	(0.14, 2.03)	0.68	(−0.13, 1.49)
Q3 (123.4)	0.13	(−0.87, 1.13)	−0.43	(−1.31, 0.45)
Q4 (150.5)	1.18	(0.09, 2.28)	0.15	(−0.81, 1.12)
Q5 (194.0)	1.89	(0.52, 3.27)	0.02	(−1.19, 1.25)
*p* value for trend		0.031		0.641

As %EI				
Q1 (median: 17.2%)		Reference		Reference
Q2 (20.7%)	−0.51	(−1.43, 0.41)	−0.24	(−1.03, 0.55)
Q3 (23.1%)	−0.26	(−1.18, 0.66)	−0.47	(−1.27, 0.33)
Q4 (25.5%)	0.09	(−0.83, 1.01)	−0.47	(−1.27, 0.34)
Q5 (29.5%)	1.51	(0.59, 2.43)	0.01	(−0.82, 0.85)
*p* value for trend		0.001		0.818

*Note*: Linear mixed effects models with repeated measurements nested within participants were fitted and regression coefficients (Coef.) reported. Model 1 was adjusted for continuous total energy intake and the time indicator. Model 2 model was additionally adjusted for sex, age groups, marital status, education, alcohol intake, physical activity, starch intake (quintiles), BMI group, history of diabetes, heart disease, hypertension and stroke, self‐rated general health, toothbrushing, fluoride toothpaste use, interdental cleaning, and dental attendance.

Abbreviations: %EI, percent of total energy intake; Q, quintiles (median value of intake).

In subsequent analysis by food groups, the intake of potatoes, roots and tubers, and wholegrains were positively associated with the baseline DMFT score whereas the intake of potato products, refined grains and pasta were inversely associated with the baseline DMFT score before adjustment for covariates (Model 1, Table 4). However, only the intake of pasta remained associated with the baseline DMFT score after adjustments (Model 2, Table 4). Adults in the highest quintile of intake of pasta had a baseline DMFT score lower by 1.31 (95% CI: −2.16, −0.46) teeth than those in the lowest quintile of intake. The interaction of pasta intake with time was not significant in Model 3 (*p* = 0.134), suggesting that the observed baseline differences in DMFT score between intake quintiles continued unchanged over the 11‐year period. The interaction of time with intake of potatoes (*p* = 0.350), potato products (*p* = 0.468), roots and tubers (*p* = 0.358), wholegrains (*p* = 0.675), refined grains (*p* = 0.323) and legumes (*p* = 0.473) were not significant either.

**TABLE 4:** Models for the association between selected food groups and 11‐year change in DMFT among Finnish adults 30 years and older (3564 observations in 1679 participants).

Food groups	Model 1		Model 2	
	Coef.	(95% CI)	Coef.	(95% CI)
Potatoes (g/day)				
Q1 (median: 67.9)		Reference		Reference
Q2 (103.5)	0.70	(−0.22, 1.63)	0.21	(−0.58, 0.99)
Q3 (139.2)	0.49	(−0.45, 1.44)	−0.01	(−0.82, 0.80)
Q4 (180.9)	1.71	(0.77, 2.66)	0.39	(−0.42, 1.21)
Q5 (241.8)	1.86	(0.82, 2.90)	0.07	(−0.83, 0.97)
*p* value for trend		<0.001		0.697

Potato products (g/day)				
Q1 (median: 0.8)		Reference		Reference
Q2 (3.4)	−1.24	(−2.15, −0.34)	−0.03	(−0.82, 0.76)
Q3 (4.5)	−1.55	(−2.48, −0.62)	−0.24	(−1.06, 0.57)
Q4 (5.9)	−2.51	(−3.46, −1.57)	−0.48	(−1.32, 0.36)
Q5 (9.4)	−3.30	(−4.27, −2.33)	−0.47	(−1.36, 0.42)
*p* value for trend		<0.001		0.172

Roots and tubers (g/day)				
Q1 (median: 13.7)		Reference		Reference
Q2 (24.8)	−0.19	(−1.11, 0.74)	−0.46	(−1.24, 0.33)
Q3 (40.6)	0.96	(0.03, 1.89)	0.06	(−0.74, 0.85)
Q4 (61.0)	0.40	(−0.55, 1.35)	−0.74	(−1.56, 0.08)
Q5 (94.0)	1.56	(0.57, 2.54)	−0.22	(−1.10, 0.65)
*p* value for trend		0.001		0.458

Legumes (g/day)				
Q1 (median: 2.8)		Reference		Reference
Q2 (6.9)	−0.55	(−1.46, 0.35)	−0.59	(−1.35, 0.17)
Q3 (9.9)	0.25	(−0.70, 1.20)	0.17	(−0.63, 0.97)
Q4 (14.6)	−0.20	(−1.15, 0.75)	−0.29	(−1.09, 0.51)
Q5 (32.7)	−0.01	(−0.99, 0.96)	−0.59	(−1.42, 0.24)
*p* value for trend		0.770		0.375

Pasta (g/day)				
Q1 (median: 2.9)		Reference		Reference
Q2 (3.0)	−0.97	(−1.85, −0.09)	−0.22	(−1.00, 0.56)
Q3 (6.3)	−1.19	(−2.06, −0.32)	−0.06	(−0.84, 0.71)
Q4 (6.4)	−3.08	(−3.92, −2.25)	−0.47	(−1.25, 0.31)
Q5 (19.1)	−4.99	(−5.87, −4.11)	−1.31	(−2.16, −0.46)
*p* value for trend		<0.001		0.007

Wholegrains (g/day)				
Q1 (median: 13.1)		Reference		Reference
Q2 (30.8)	1.00	(0.08, 1.92)	0.43	(−0.35, 1.21)
Q3 (57.2)	0.76	(−0.16, 1.69)	−0.66	(−1.45, 0.13)
Q4 (82.7)	2.43	(1.47, 3.38)	0.17	(−0.66, 1.01)
Q5 (122.5)	1.57	(0.59, 2.55)	−0.50	(−1.36, 0.35)
*p* value for trend		<0.001		0.168

Refined grains (g/day)				
Q1 (median: 48.2)		Reference		Reference
Q2 (73.1)	−0.15	(−1.08, 0.78)	0.43	(−0.36, 1.22)
Q3 (98.5)	−0.55	(−1.50, 0.41)	0.23	(−0.58, 1.04)
Q4 (126.2)	−0.75	(−1.75, 0.25)	0.08	(−0.77, 0.93)
Q5 (178.5)	−1.38	(−2.52, −0.24)	0.20	(−0.78, 1.18)
*p* value for trend		0.013		0.981

*Note*: Linear mixed effects models with repeated measurements of DMFT nested within participants were fitted and regression coefficients (Coef.) reported. Model 1 was adjusted for continuous total energy intake and the time indicator. Model 2 was also adjusted for sex, marital status, education, alcohol intake, physical activity, BMI group, history of diabetes, heart disease, hypertension and stroke, self‐rated general health, toothbrushing, fluoride toothpaste use, dental flossing, and dental attendance.

Abbreviation: Q, quintiles.

In supplemental analysis, we found that adults in the study sample were more likely to be female, younger, and more educated and have better behaviors and health status than adults without follow‐up data on dental caries and adults with follow‐up data on dental caries but no data on diet or covariates (Table S1). Second, neither the intake of total starch nor that of starch‐rich foods was associated with the DFT score. Third, adjusting only for common causes of exposure and outcome did not change the findings of the study. Total starch intake was not associated with the DMFT score whereas the intake of pasta was inversely associated with the baseline DMFT score (Table S2). Finally, a power calculation indicated that our study had 82.3% power to reject the null hypothesis of no differences in the 11‐year change in the DMFT score observed between the highest (0.56 ± 1.75, *n* = 336) and lowest quintiles (0.98 ± 2.02 teeth, *n* = 335) of baseline starch intake, using a two‐sided two‐sample unequal‐variance *t*‐test with a significance level of 0.05.

## DISCUSSION

This longitudinal study found that neither the amount nor the type of starch intake was associated with changes in dental caries over 11 years. Almost all associations observed in crude models were fully accounted for by measured covariates, highlighting the crucial role of common determinants of starch intake and dental caries (such as demographic and socioeconomic factors). Although the DMFT score is expected to increase across timepoints given the progressive nature of the disease, the DMFT increment of 0.80 over 11 years was at the lower end of caries progression rates among adults [27].

Contrary to our hypothesis, foods high in RDS (e.g. potatoes, tubers and roots and refined grains) were not associated with changes in dental caries. This is in line with a previous 3‐year longitudinal study among US children reporting that the intake of unprocessed starch (e.g. boiled potato, bread, rice) was not associated with caries increment [28]. That said, we could not confirm their finding that the frequent intake of processed starch (e.g. potato crisps) between meals was associated with caries increment [28]. Although our food group of potato products, which included French fries and potato chips, was comparable to the group of processed starch used in the previous study, its consumption was low among our participants and probably lower than among children. Despite their potential benefits on general health [8], foods high in SDS or RS (e.g. wholegrains and legumes) were not associated with changes in dental caries. Of the seven food groups evaluated, only pasta intake was associated with dental caries, although that association was only observed at baseline. Pasta is a starch‐rich food with a low glycemic index (i.e. it induces a small postprandial glycemic response) and is one of the main components in the Mediterranean diet [29, 30]. Any potential protective effect of pasta could be attributed to their lower digestibility (i.e. making them inaccessible to oral bacteria) and greater satiety after ingestion. Although semolina (the main ingredient in pasta) is regarded as a source of RDS, its starch structure can be altered during processing and cooking. The pasta making process creates a highly compact matrix that reduces the fraction of RDS of native starch in semolina. This matrix is further strengthened during cooking and preserved even after prolonged cooking times [30]. Moreover, individuals felt less hungry, fuller, more satisfied, and wanted to eat less following pasta and potato‐based meals [31, 32], which implies that individuals could skip dessert or avoid snacking between meals. Pasta intake could also be a marker of a different (healthier) lifestyle. As such, this finding must be taken with caution. As already explained, pasta intake was associated with dental caries cross‐sectionally (baseline only) rather than longitudinally (changes in dental caries). What is more, we were unable to replicate this finding with other common sources of RS (i.e. legumes) available in our analysis. Therefore, this finding awaits confirmation from further longitudinal studies.

The strengths of this study include the large sample size, the length of the follow‐up (three caries assessments over 11 years), the assessment of both intra‐ and inter‐examiner reliability for caries examinations and the comprehensive set of covariates included. However, the study has some limitations worth discussing. First, our data covered the period 2000–2011. Although some may question whether our findings are still relevant today, we argue that the role of dietary factors in caries development (e.g. free sugars) has not changed since it was first identified and continues to be observed despite improvements in diet and widespread caries preventive measures. Second, as is common in longitudinal studies, the sample was reduced due to losses to follow‐up and missing data. Retained adults were more likely to be younger, more educated, and have better behaviors and health status. This means that the present findings cannot be generalized beyond the study sample. Third, our dietary assessment was based on the single administration of a validated FFQ (wave 1), which cannot capture changes in food consumption over time. While FFQs measure habitual dietary intake over an extended period, they lack the level of detail captured with more intensive assessment methods such as food recalls or diaries [33]. However, the original validation study showed that the energy intake recorded in the FFQ was 95% of that in 7‐day food records [19], thus supporting the accuracy of our dietary assessment. Also, data on timing of consumption (i.e. within and between meals) and properties of starch‐rich foods (i.e. retentiveness) were not available. Third, dental caries was summarized using the DMFT score, which is not without criticisms. One criticism is the reliability of identifying missing teeth due to caries (as opposed to other reasons such as periodontitis, dental traumam, or orthodontics) among adults. However, we found similar results when using the DFT score instead of the full DMFT score.

The findings of this study suggest that the amount of starch intake may not be a direct determinant of dental caries risk, and the focus should be shifted towards understanding the differential effects of starch types (if any). Analysis of food groups allows considering country‐specific topics such as local availability of certain foods and dietary preferences or traditions. Even if there are no benefits to dental status from consuming SDS/RS, dentists should promote the consumption of wholegrains, vegetables, fruits, and legumes as main carbohydrate sources given their known benefits on general health [8, 9, 10].

Stronger methods to determine starch digestibility in epidemiologic studies are also needed. Starch comes in multiple forms, depending on methods for storage, cooking, processing, mixing with other foods and serving temperature, which alters its structure and properties [2, 7]. Currently, the well‐known classification of starch into RDS, SDS, and RS [1, 5] cannot be fully operationalized in epidemiologic studies because such a disaggregation of starch is unavailable in food composition tables [14]. Thus, researchers resort to use broadly defined food sources as proxies for types of starch. Incorporating the glycemic index of specific foods or the glycemic load as a factor in future studies could provide valuable insights. By refining the classification of starches and employing more precise dietary assessment methods (e.g. multiple‐pass 24‐h recalls) repeated over time, researchers can gain a deeper understanding of the relationship between starch intake and dental caries. Finally, it is important to acknowledge that starch is not consumed in isolation but rather as part of a complex dietary pattern. Future research should consider the synergistic and antagonistic effects of various dietary components on oral diseases.

In conclusion, this study found no association between the amount and type of starch intake and changes in dental caries among Finnish adults over an 11‐year period.

## CONFLICT OF INTEREST STATEMENT

The authors declare no conflicts of interest.

## Supporting information


**Data S1.** Supporting Information.
