# Supplementary material for: Starch intake and changes in dental caries among adults: A longitudinal study in Finland
Source: J Public Health Dent. 2024 Nov 16;85(1):29–39. doi: 10.1111/jphd.12650 (PMC11927949; doi:10.1111/jphd.12650)
Supplement: Supplementary file 1 — Data S1. Supporting Information. [file JPHD-85-29-s001.docx]

**Supplemental files for manuscript entitled “Starch intake and changes in dental caries among adults: A longitudinal study in Finland”**

**Authors:**

Jangda FH, Suominen AL, Lundqvist A, Männistö S, Golkari A, Bernabé E

**Table of contents**

**Figure S1.** Directed acyclic graph describing the relationship among baseline starch intake (exposure), 11-year change in dental caries (outcome) and the set of confounders.

**Table S1.** Comparison of the characteristics of adults without follow-up data on dental caries (group 1), adults with follow-up data on dental caries but no data on diet or covariates (group 2) and adults in the study sample (group 3).

**Table S2.** Models for the association of starch amount and type with 11-year change in DMFT among Finnish adults 30 years and older (3564 observations in 1679 participants), adjusting for common causes of starch intake and dental caries only.

**
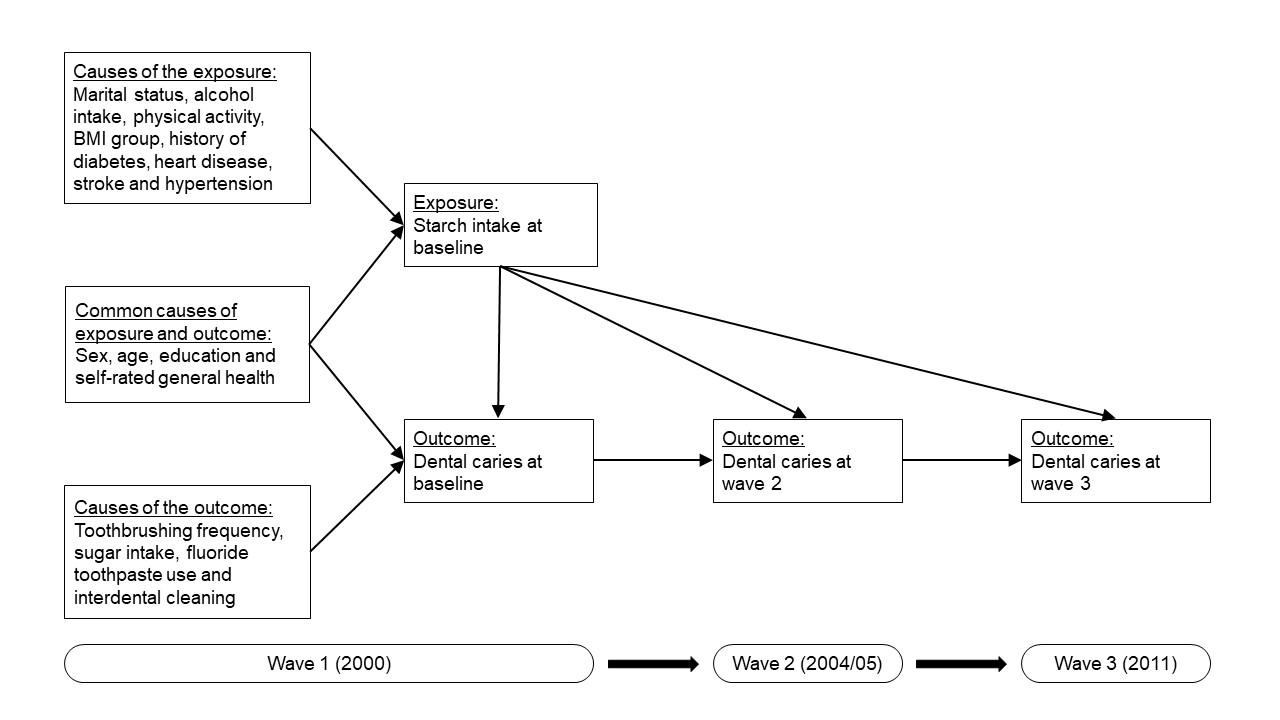
**

**Figure S1.** Directed acyclic graph describing the relationship among baseline starch intake (exposure), 11-year change in dental caries (outcome) and the set of confounders.

**Table S1.** Comparison of the characteristics of adults without follow-up data on dental caries (group 1), adults with follow-up data on dental caries but no data on diet or covariates (group 2) and adults in the study sample (group 3).

| **Baseline characteristics** | | **Group 1** | | **Group 2** | | **Group 3** | | **Group 1 vs. 3**^a^ | **Group 2 vs. 3**^a^ |
| --- | --- | --- | --- | --- | --- | --- | --- | --- | --- |
|  |  | **n** | **%** | **n** | **%** | **n** | **%** |  |  |
| *Sex* | |  |  |  |  |  |  | 0.08 | 0.13 |
|  | Male | 1690 | 48.2 | 107 | 50.7 | 740 | 44.1 |  |  |
|  | Female | 1819 | 51.8 | 104 | 49.3 | 939 | 55.9 |  |  |
| *Age groups* | |  |  |  |  |  |  | 0.34 | 0.22 |
|  | 30-39 years | 867 | 24.7 | 58 | 27.5 | 498 | 29.7 |  |  |
|  | 40-49 years | 944 | 26.9 | 54 | 25.6 | 487 | 29.0 |  |  |
|  | 50-59 years | 759 | 21.6 | 57 | 27.0 | 423 | 25.2 |  |  |
|  | 60-69 years | 505 | 14.4 | 24 | 11.4 | 209 | 12.5 |  |  |
|  | 70+ years | 434 | 12.4 | 18 | 8.5 | 62 | 3.7 |  |  |
| *Education* | |  |  |  |  |  |  | 0.31 | 0.16 |
|  | Basic | 1290 | 36.9 | 63 | 31.0 | 402 | 23.9 |  |  |
|  | Secondary | 1207 | 34.5 | 65 | 32.0 | 599 | 35.7 |  |  |
|  | Higher | 998 | 28.6 | 75 | 37.0 | 678 | 40.4 |  |  |
| *Marital status* | |  |  |  |  |  |  | 0.12 | 0.06 |
|  | Cohabiting | 2474 | 70.7 | 149 | 73.4 | 1275 | 75.9 |  |  |
|  | Living alone | 1025 | 29.3 | 54 | 26.6 | 404 | 24.1 |  |  |
| *Physical activity* | |  |  |  |  |  |  | 0.11 | 0.07 |
|  | Sedentary | 1335 | 38.9 | 69 | 37.5 | 611 | 36.4 |  |  |
|  | Low | 997 | 29.1 | 62 | 33.7 | 531 | 31.6 |  |  |
|  | Sufficient | 982 | 28.7 | 44 | 23.9 | 454 | 27.0 |  |  |
|  | Ideal | 114 | 3.3 | 9 | 4.9 | 83 | 4.9 |  |  |
| *Alcohol consumption* | |  |  |  |  |  |  | 0.15 | 0.21 |
|  | No use | 526 | 15.3 | 33 | 17.1 | 173 | 10.3 |  |  |
|  | Moderate use | 2201 | 64.1 | 118 | 61.1 | 1158 | 69.0 |  |  |
|  | Risk use | 705 | 20.5 | 42 | 21.8 | 348 | 20.7 |  |  |
| *BMI group* | |  |  |  |  |  |  | 0.09 | 0.14 |
|  | Normal | 1319 | 37.7 | 74 | 35.2 | 700 | 41.7 |  |  |
|  | Overweight | 1405 | 40.1 | 87 | 41.4 | 659 | 39.3 |  |  |
|  | Obese | 777 | 22.2 | 49 | 23.3 | 320 | 19.1 |  |  |
| *Diabetes* | |  |  |  |  |  |  | 0.10 | 0.13 |
|  | No | 3323 | 95.1 | 192 | 94.6 | 1630 | 97.1 |  |  |
|  | Yes | 172 | 4.9 | 11 | 5.4 | 49 | 2.9 |  |  |
| *Hearth disease* | |  |  |  |  |  |  | 0.11 | 0.07 |
|  | No | 2736 | 78.2 | 162 | 79.8 | 1385 | 82.5 |  |  |
|  | Yes | 763 | 21.8 | 41 | 20.2 | 294 | 17.5 |  |  |
| *Hypertension* | |  |  |  |  |  |  | 0.10 | 0.10 |
|  | No | 2445 | 69.9 | 160 | 78.8 | 1252 | 74.6 |  |  |
|  | Yes | 1051 | 30.1 | 43 | 21.2 | 427 | 25.4 |  |  |
| *Stroke* | |  |  |  |  |  |  | 0.05 | 0.05 |
|  | No | 3428 | 98.1 | 199 | 98.0 | 1657 | 98.7 |  |  |
|  | Yes | 68 | 2.0 | 4 | 2.0 | 22 | 1.3 |  |  |
| *Self-rated general health* | |  |  |  |  |  |  | 0.27 | 0.25 |
|  | Poor | 371 | 10.6 | 23 | 11.5 | 82 | 4.9 |  |  |
|  | Moderate | 919 | 26.3 | 42 | 21.0 | 357 | 21.3 |  |  |
|  | Good | 2201 | 63.1 | 135 | 67.5 | 1240 | 73.9 |  |  |
| *Toothbrushing* | |  |  |  |  |  |  | 0.23 | 0.29 |
|  | Twice or more daily | 1991 | 59.2 | 76 | 57.1 | 1153 | 68.7 |  |  |
|  | Once daily | 1089 | 32.4 | 44 | 33.1 | 459 | 27.3 |  |  |
|  | Less than daily | 282 | 8.4 | 13 | 9.8 | 67 | 4.0 |  |  |
| *Fluoride toothpaste use* | |  |  |  |  |  |  | 0.17 | 0.05 |
|  | Daily | 2899 | 87.7 | 107.0 | 91.5 | 1555 | 92.6 |  |  |
|  | Less than daily | 278 | 8.4 | 7.0 | 6.0 | 83 | 4.9 |  |  |
|  | Never | 130 | 3.9 | 3.0 | 2.6 | 41 | 2.4 |  |  |
| *Interdental cleaning* | |  |  |  |  |  |  | 0.19 | 0.41 |
|  | Daily | 302 | 9.1 | 5 | 4.1 | 200 | 11.9 |  |  |
|  | Less than daily | 1165 | 35.1 | 38 | 31.2 | 695 | 41.4 |  |  |
|  | Never | 1851 | 55.8 | 79 | 64.8 | 784 | 46.7 |  |  |
| *Dental attendance* | |  |  |  |  |  |  | 0.12 | 0.18 |
|  | For check-ups | 1925 | 57.2 | 73 | 54.1 | 1055 | 62.8 |  |  |
|  | Only when in trouble | 1441 | 42.8 | 62 | 45.9 | 624 | 37.2 |  |  |

^a^ Standardised difference was used to compare groups. Values greater than 0.10 indicate covariate imbalance between groups.

**Table S2.** Models for the association of starch amount and type with 11-year change in DMFT among Finnish adults 30 years and older (3564 observations in 1679 participants), adjusting for common causes of starch intake and dental caries only.

|  | | **Coef.** | **(95% CI)** | **P value for interaction with time** |
| --- | --- | --- | --- | --- |
| *Starch intake (g/day)* | |  |  | *0.343* |
|  | Q1 (median: 71.6) |  | Reference |  |
|  | Q2 (101.4) | 0.67 | (-0.14, 1.47) |  |
|  | Q3 (123.4) | -0.53 | (-1.38, 0.32) |  |
|  | Q4 (150.5) | 0.26 | (-0.67, 1.19) |  |
|  | Q5 (194.0) | 0.08 | (-1.10, 1.26) |  |
|  | *P value for trend* |  | *0.779* |  |
| *Starch intake (%EI)* | |  |  | *0.320* |
|  | Q1 (median: 17.2%) |  | Reference |  |
|  | Q2 (20.7%) | -0.26 | (-1.04, 0.52) |  |
|  | Q3 (23.1%) | -0.53 | (-1.31, 0.25) |  |
|  | Q4 (25.5%) | -0.49 | (-1.27, 0.30) |  |
|  | Q5 (29.5%) | -0.04 | (-0.83, 0.76) |  |
|  | *P value for trend* |  | *0.715* |  |
| *Potatoes (g/day)* | |  |  | *0.363* |
|  | Q1 (median: 67.9) |  | Reference |  |
|  | Q2 (103.5) | 0.16 | (-0.63, 0.95) |  |
|  | Q3 (139.2) | -0.07 | (-0.87, 0.74) |  |
|  | Q4 (180.9) | 0.37 | (-0.44, 1.18) |  |
|  | Q5 (241.8) | 0.09 | (-0.80, 0.99) |  |
|  | *P value for trend* |  | *0.646* |  |
| *Potato products (g/day)* | |  |  | *0.470* |
|  | Q1 (median: 0.8) |  | Reference |  |
|  | Q2 (3.4) | -0.03 | (-0.82, 0.76) |  |
|  | Q3 (4.5) | -0.30 | (-1.12, 0.51) |  |
|  | Q4 (5.9) | -0.48 | (-1.32, 0.36) |  |
|  | Q5 (9.4) | -0.45 | (-1.34, 0.43) |  |
|  | *P value for trend* |  | *0.179* |  |
| *Roots and tubers (g/day)* | |  |  | *0.351* |
|  | Q1 (median: 13.7) |  | Reference |  |
|  | Q2 (24.8) | -0.44 | (-1.23, 0.34) |  |
|  | Q3 (40.6) | 0.07 | (-0.73, 0.86) |  |
|  | Q4 (61.0) | -0.72 | (-1.54, 0.09) |  |
|  | Q5 (94.0) | -0.11 | (-0.98, 0.76) |  |
|  | *P value for trend* |  | *0.606* |  |
| *Legumes (g/day)* | |  |  | *0.479* |
|  | Q1 (median: 2.8) |  | Reference |  |
|  | Q2 (6.9) | -0.67 | (-1.44, 0.09) |  |
|  | Q3 (9.9) | 0.16 | (-0.65, 0.96) |  |
|  | Q4 (14.6) | -0.31 | (-1.12, 0.49) |  |
|  | Q5 (32.7) | -0.51 | (-1.34, 0.32) |  |
|  | *P value for trend* |  | *0.502* |  |
| *Pasta (g/day)* | |  |  | *0.144* |
|  | Q1 (median: 2.9) |  | Reference |  |
|  | Q2 (3.0) | -0.23 | (-1.01, 0.55) |  |
|  | Q3 (6.3) | -0.16 | (-0.94, 0.61) |  |
|  | Q4 (6.4) | -0.60 | (-1.37, 0.18) |  |
|  | Q5 (19.1) | -1.42 | (-2.26, -0.57) |  |
|  | *P value for trend* |  | *0.002* |  |
| *Wholegrains (g/day)* | |  |  | *0.680* |
|  | Q1 (median: 13.1) |  | Reference |  |
|  | Q2 (30.8) | 0.46 | (-0.32, 1.25) |  |
|  | Q3 (57.2) | -0.63 | (-1.43, 0.16) |  |
|  | Q4 (82.7) | 0.26 | (-0.57, 1.09) |  |
|  | Q5 (122.5) | -0.52 | (-1.37, 0.33) |  |
|  | *P value for trend* |  | *0.176* |  |
| *Refined grains (g/day)* | |  |  | *0.322* |
|  | Q1 (median: 48.2) |  | Reference |  |
|  | Q2 (73.1) | 0.35 | (-0.44, 1.14) |  |
|  | Q3 (98.5) | 0.16 | (-0.65, 0.98) |  |
|  | Q4 (126.2) | 0.00 | (-0.85, 0.86) |  |
|  | Q5 (178.5) | 0.08 | (-0.90, 1.06) |  |
|  | *P value for trend* |  | *0.861* |  |

Q: Quintiles.

Linear mixed effects models with repeated measurements of DMFT nested within participants were fitted and regression coefficients (Coef.) reported. Models were adjusted for energy intake (continuous) the time indicator and common causes of starch intake and dental caries (sex, age groups, education and self-rated general health).
